# A Comprehensive and Rapid Quality Evaluation Method of Traditional Chinese Medicine Decoction by Integrating UPLC-QTOF-MS and UFLC-QQQ-MS and Its Application

**DOI:** 10.3390/molecules24020374

**Published:** 2019-01-21

**Authors:** Yinfang Chen, Riyue Yu, Li Jiang, Qiyun Zhang, Bingtao Li, Hongning Liu, Guoliang Xu

**Affiliations:** 1 College of Pharmacy, Hunan University of Chinese Medicine, Changsha 410208, China; 20091031@jxutcm.edu.cn; 2 College of Pharmacy, Jiangxi University of Traditional Chinese Medicine, Nanchang 330004, China; 19820354@jxutcm.edu.cn; 3 Key Laboratory of Pharmacology of Traditional Chinese Medicine in Jiangxi, Jiangxi University of Traditional Chinese Medicine, Nanchang 330004, China; 4 Research Center for Differention and Development of TCM Basic Theory, Jiangxi University of Traditional Chinese Medicine, Nanchang 330004, China; jiangli1009@126.com (L.J.); 20060874@jxutcm.edu.cn (Q.Z.); 20151008@jxutcm.edu.cn (B.L.); 5 Jiangxi Province Key Laboratory of TCM Etiopathogenisis, Jiangxi University of Traditional Chinese Medicine, Nanchang 330004, China

**Keywords:** traditional Chinese medicine decoction, quality evaluation, UPLC-QTOF-MS, UFLC-QQQ-MS, ShenFu prescription decoction

## Abstract

Decoction is one of the oldest forms of traditional Chinese medicine and it is widely used in clinical practice. However, the quality evaluation and control of traditional decoction is a challenge due to the characteristics of complicated constituents, water as solvent, and temporary preparation. ShenFu Prescription Decoction (SFPD) is a classical prescription for preventing and treating many types of cardiovascular disease. In this article, a comprehensive and rapid method for quality evaluation and control of SFPD was developed, via qualitative and quantitative analysis of the major components by integrating ultra-high-performance liquid chromatography equipped with quadrupole time-of-flight mass spectrometry and ultra-fast-performance liquid chromatography equipped with triple quadrupole mass spectrometry. Consequently, a total of 39 constituents were tentatively identified in qualitative analysis, of which 21 compounds were unambiguously confirmed by comparing with reference substances. We determined 13 important constituents within 7 min by multiple reaction monitoring. The validated method was applied for determining five different proportion SFPDs. It was found that different proportions generated great influence on the dissolution of constituents. This may be one of the mechanisms for which different proportions play different synergistic effects. Therefore, the developed method is a fast and useful approach for quality evaluation of SFPD.

## 1. Introduction

Decoction of traditional Chinese medicine (TCM) is a liquid dosage form, which is prepared by soaking and decocting the slices or coarse granules of medicinal materials with water and removing the dregs to extract a solution [1,2,3]. It is one of the earliest and most widely used dosage forms in Chinese medical practice and has existed for thousands of years [4]. Not only is decoction a relatively fast and cheap process that is readily available to patients, but also the flexible prescription can satisfy the needs of TCM treatment according to syndrome differentiation [5], Nevertheless, how effective the drug prescription is, depends on the quality of decoction [6]. Furthermore, decoction of TCM is a complex system consisting of many components. Therefore, it is a challenge for quality evaluation and control of traditional decoction.

ShenFu Prescription Decoction (SFPD), comprised of Hongshen (steamed roots of *Ginseng Radix et Rhizoma*) and Fuzi(Heishunpian, processed lateral roots of *Aconitum carmichaeli Debx*) [7], is a rather important classical prescription of replenishing Qi and warming Yang, and is recorded in dozens of ancient medical books. Up to now, it is still widely used in clinical practice for preventing and treating many types of cardiovascular disease [8,9,10,11]. However, there are several different dosages ratios of Hongshen and Fuzi for different symptoms, such as 5:1, 3:2, 2:1, 1:2, and so on. Therefore, it is essential to clarify the chemical compositions and develop a fast and powerful approach for quality evaluation and control to ensure the efficacy of SFPD. Previous research has confirmed that the curative effect of SFPD is an integrative effect of ginsenosides and alkaloids [12,13,14]. Several methods for component analysis and determination of major constituents in ShenFu injection and their serum pharmacochemistry have been reported by using HPLC-PAD [15], UPLC-Q-TOF-MS [16,17], and HILIC-RPLC-MS/MS technologies [14]. Few papers have focused specifically on the decoction of ShenFu, either investigating pharmacochemistry [12] or just quantitative analysis for several constituents [18]. Therefore, it is still necessary to develop a set of comprehensive and rapid quality evaluation methods for qualitative analysis and quantitative analysis of the major components in SFPD simultaneously.

However, the conventional column chromatography combined with diode array detector (DAD) [19] or evaporative light scattering detector (ELSD) [20] detector do not satisfy the needs required for accurate and rapid analysis of complex components due to low peak capacity, time-consumption, and low sensitivity. Currently, the high-throughput and high-resolution UPLC-Q-TOF-MS, and ultra-fast and high-sensitivity UFLC-MS/MS systems easily achieve this goal, and are thus are employed as powerful tools for investigating the chemical constituents in complex Chinese medicine [21,22].

**Figure 1:**
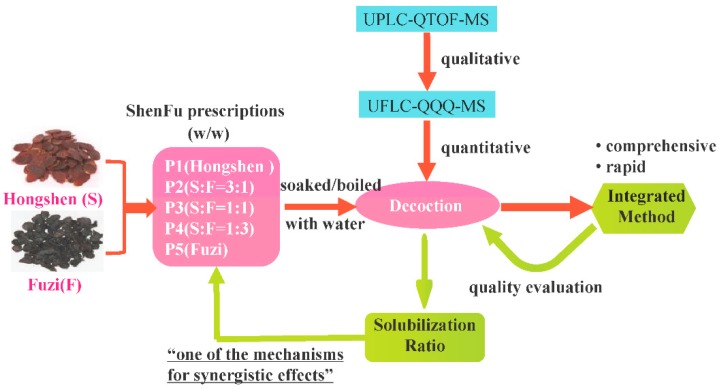
The flow chart illustrates the overall strategy for research.

In this article, a sensitive and high-throughput UPLC-Q-TOF-MS method was established to analyze and identify the overall constituents of SFPD comprehensively. According to the global constituent profiles, the biological activities of the major constituents [23,24], and their abundance in pre-test samples, seven aconitum alkaloids (benzoylmesaconine (BMA), benzoylhypaconine (BHA), benzoylaconine (BAC), mesaconitine (MA), hypaconitine (HA), aconitine (AC), Fuziline (FZL)), and six ginsenosides(Rb_1_, Rb_3_, Rd, Re, Rg_1_, Ro) were selected as the quality markers for developing a quantitative analysis method by UFLC-QQQ-MS. Following this, the validated method was applied so as to determine the contents of different proportions of SFPD and further investigate the influence on dissolution of constituents generated by different ratios of Hongshen and Fuzi. During this process, a concept of solubilization ratio was introduced to present the effect and to strive to illuminate the potential mechanism of different proportion prescriptions that produce different synergistic effects from a material basis. The flow chart illustrating the strategy is shown in Figure 1.

## 2. Results and Discussion

### 2.1. Optimization of UPLC-QTOF-MS and UFLC-QQQ-MS Conditions

It is necessary to optimize the analysis conditions for identifying as many constituents as possible in qualitative analysis of SFPD. Firstly, several columns, such as Agilent Eclipse Plus C18 column (100 mm × 2.1 mm, 3.5 μm), Thermo Scientific Hypersil GOLD C18 column (150 mm × 2.1 mm, 3.5 μm), Waters ACQUITY UPLC BEH C18 column (50 mm × 2.1 mm, 1.7 μm), and Thermo Scientific Hypersil GOLD C18 column (100 mm × 2.1 mm, 1.9 μm) were investigated. The result showed that Thermo Scientific Hypersil GOLD C18 column (100 mm × 2.1 mm, 1.9 μm) provided a better separation for most of the constituents in 30 min. Methanol and acetonitrile were compared as an organic phase and acetonitrile showed a better separation capability. Moreover, when formic acid was added in aqueous phase, the responses and shapes of most chromatographic peaks improved significantly. The 0.1% formic acid was tested to be proper. Several column temperatures (25 °C, 30 °C, 35 °C, and 40 °C), flow rates, and different elution programs were also examined in advance. Finally, mobile phase was composed of 0.1% formic acid in water (A) and acetonitrile (B) with a gradient program as follows: 5% (B) in 0 to 2 min, 5% to 100% (B) in 2 to 30 min and delivered at a flow rate of 0.2 mL/min. The column temperature was operated at 35 °C.

While in quantitative analysis, the column, mobile phase, and other chromatographic conditions were also tested beforehand to achieve a good separation and fast detection for all the analytes. Consequently, the mobile phase was composed of 0.1% formic acid in water (A) and acetonitrile (B) with a very fast gradient program as follows: 5% to 40% (B) in 0 to 3 min, 40% (B) in 3 to 5 min, 40% to 80% (B) in 5 to 5.5 min, 80% (B) in 5.5 to 7 min, 80% to 5% (B) in 7 to 7.1 min, 5% (B) in 7.1 to 9 min. The flow rate was set at 0.3 mL/min and column temperature was operated at 40 °C.

### 2.2. Identification of Chemical Constituents of SFPD by UPLC-QTOF-MS

**Figure 2:**
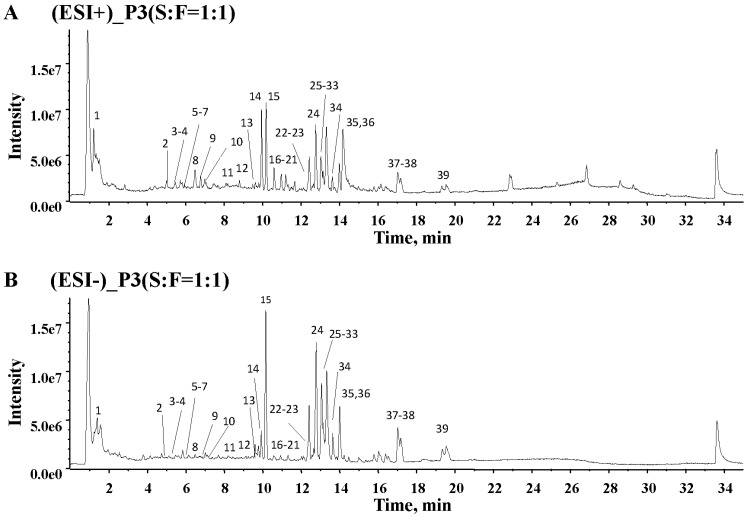
Representative total ion chromatograms of the ShenFu Prescription Decoction (SFPD) by UPLC-QTOF-MS. (**A**) Total ion chromatograms (TIC) of P3 in positive ion mode; (**B**) TIC of P3 in negative ion mode.

In order to obtain more comprehensive information, total ion chromatograms (TIC) of five SFPDs were collected in both positive and negative mode. There are obvious quantitative differences of chemical composition in different proportions of SFPD from the TICs (Figure S1). The representative TICs of P3 (S:F = 1:1) in positive and negative ion modes were acquired for identification the chemical constituents of SFPD as shown in Figure 2.

An in-house constituent library including the major known constituents of Hongshen and Fuzi was imported into the Peak View Software TM V.1.2 to accomplish constituent identification from the representative TICs. The preliminary identification results were further verified by accurate masses and fragment ions reported in the literature. Ultimately, a total of 39 constituents were identified or tentatively characterized, of which 17 were from Hongshen and 22 compounds were from Fuzi. The detailed results are shown in Table 1. Moreover, 21 compounds were unambiguously identified and confirmed by comparing the retention time, mass spectrum (MS) information, and MS/MS fragmental ions with their reference standards. The other compounds were tentatively defined by comparing their exact masses, MS/MS fragmental ions, and retention behaviors with previous studies.

### 2.3. Quantitative Determination of the Major Constituents in SFPD by UFLC-QQQ-MS/MS

**Figure 3:**
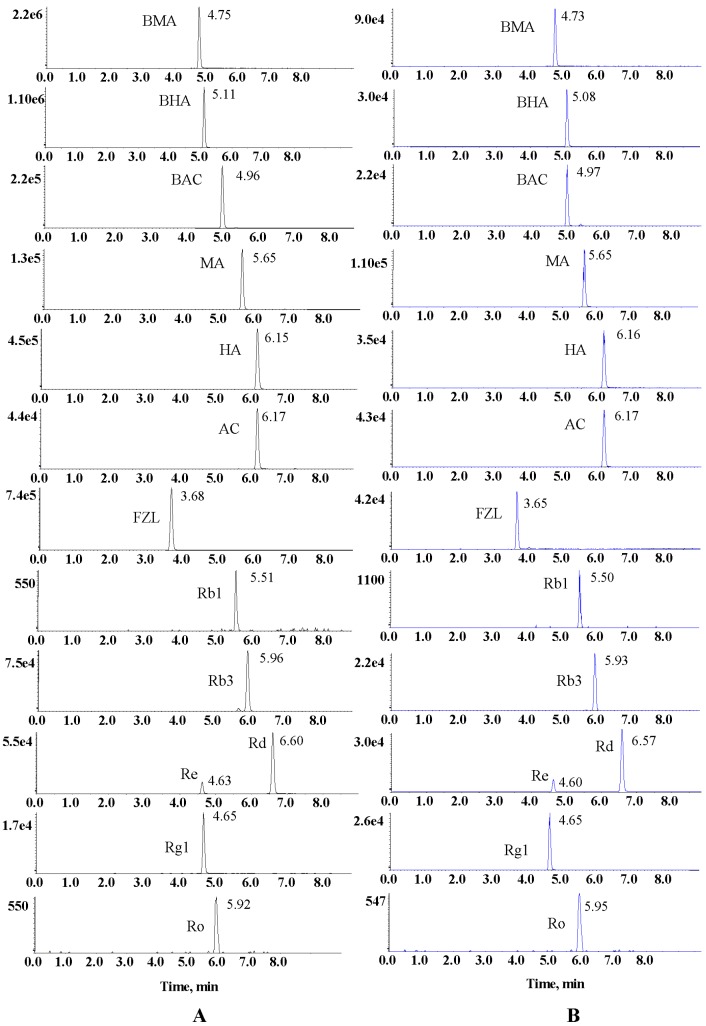
The multiple reaction monitoring (MRM) chromatograms of 13 analytes by UFLC-QQQ-MS/MS. (**A**) 13 analytes in sample solution; (**B**) 13 analytes in reference solution.

Alkaloids and ginsenosides are the main active components in SFPD. In order to achieve rapid quality control, seven alkaloids and six ginsenosides were analyzed simultaneously in this study. The responses of all the analytes were evaluated both in positive and negative ion mode beforehand. Finally, all the analytes were detected with stable and strong MS signal in positive mode. Multiple reaction monitoring (MRM) was employed to increase specificity and sensitivity of quantification analysis. The MRM pairs comprising of precursor and product ions for each analyte were investigated by infusing the single standard solution into the mass spectrometer directly in advance. The selected MRM pairs and optimum collision energy are presented in Table 2. For better ionization, 0.1% formic acid was added to mobile phase. The 13 analytes were detected simultaneously within 7 min. The MRM chromatograms of 13 analystes are shown in Figure 3. The retention time for BMA, BHA, BAC, MA, HA, AC, FZL, Rb_1_, Rb_3_, Rd, Re, Rg_1_, and Ro were 4.75, 5.11, 4.96, 5.65, 6.15, 6.17, 3.68, 5.51, 11.37, 5.96, 6.60, 4.63, 4.65, and 5.92 min, respectively.

### 2.4. Linearity and Sensitivity

The calibration curves of seven alkaloids and six ginsenosides were fitted with coefficients of determination greater than 0.99. The linear ranges were set as 0.01–50 ng/mL for BHA, BAC, HA, and AC, 0.05 to 50 ng/mL for BMA, 0.01 to 25 ng/mL for MA, and FZL, 2.5 to 312.5 ng/mL for Rb_1_, Rb_3_, and Rg_1_, 25 to 6250 ng/mL for Rd, 2.5 to 156.2 ng/mL for Re, and 5.0 to 312.5 ng/mL for Ro, respectively, according to the approximate concentrations of the sample. The limit of detections (LODs) of seven alkaloids and six ginsenosides were 0.003 ng/mL and 1.0 ng/mL, respectively. The limit of quantifications (LOQs) of seven alkaloids and six ginsenosides were 0.01 ng/mL and 2.5 ng/mL, respectively. The concrete values are listed in Table 3. The excellent linearity with wide ranges and low LOQs demonstrates that this method can be employed for determining many kinds of samples effectively, even serum samples.

### 2.5. Precision, Stability, Repeatability, and Recovery

The intraday and interday precisions were validated by mixing standard solutions with three concentration levels. The RSDs of intra- and interday precisions were less than 6.87% and 10.93%, respectively. The stability and reproducibility were evaluated by sample solutions and the RSDs were less than 7.35% and 10.13%, respectively. The detailed data are listed in Table 4. The accuracy of the developed method was verified by a recovery test. The recoveries of 13 reference substances varied from 95.14% to 106.43% (RSDs ≤ 7.00%), as shown in Table 5. These results indicate the established method is accurate, stable, and reproducible.

### 2.6. Results of Sample Analysis

The validated method was subsequently applied to investigate the contents of the 13 constituents in five SFPD samples (P1(only Hongshen), P2 (S:F = 3:1, *w*/*w*), P3 (S:F = 1:1, *w*/*w*), P4 (S:F = 1:3, *w*/*w*), and P5 (only Fuzi)) based on their respective calibration curves summarized in Table 3. Thus, P1 only included ginsenosides, while P5 consisted solely of alkaloids. Theoretically, the contents of ginsenosides and alkaloids in P2, P3, and P4 were in a certain proportion to P1 and P5. However, it is not the case. Therefore, a concept of solubilization ratio was introduced to present the effect. The solubilization ratio of 13 analytes in P2, P3, and P4 were calculated via comparing the contents in P1 or P5. The calculated formula was as follows: Solubilization ratio (%) = (detected amount − theoretical amount)/ theoretical amount × 100. Theoretical amounts for alkaloids in P2, P3, and P4 were calculated according to detected amounts in P5 (F) and theoretical amounts for ginsenosides in P2, P3, and P4 were calculated according to the detected amount in P1 (S). The detailed data and results are listed in Table 6. For most constituents, the contents did not increase or decrease proportionately in different ratio prescriptions, but generated solubilization effect or dissolution-inhibited effect, which was believed to be one of the main mechanisms of the synergistic effect of Hongshen and Fuzi. The mechanism of interaction effect of component dissolution has been regarded as an important basis for the prescriptions of TCM [28,29].

## 3. Materials and Methods

### 3.1. Materials and Reagents

**Figure 4:**
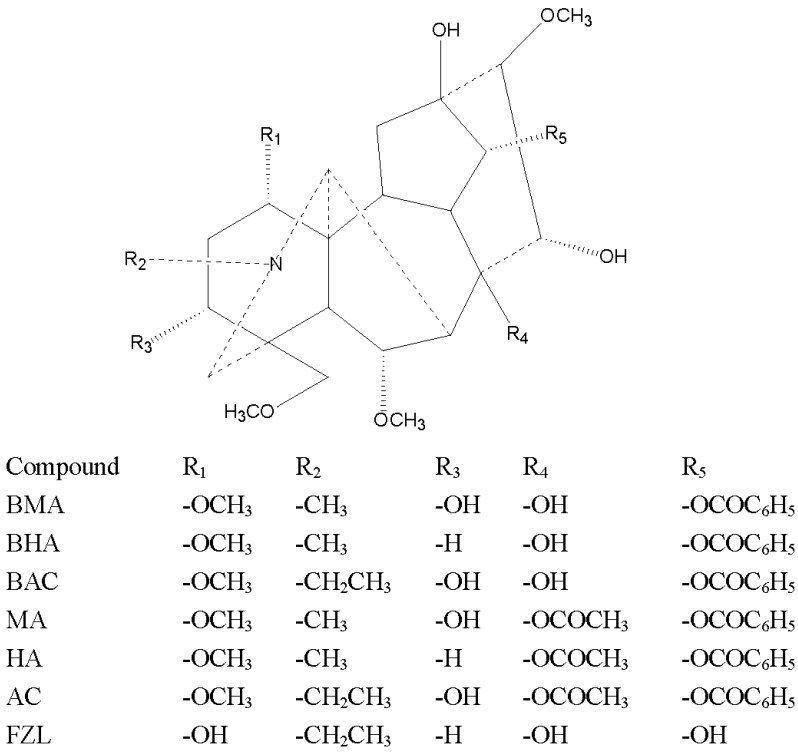
Chemical structures of alkaloids.

**Figure 5:**
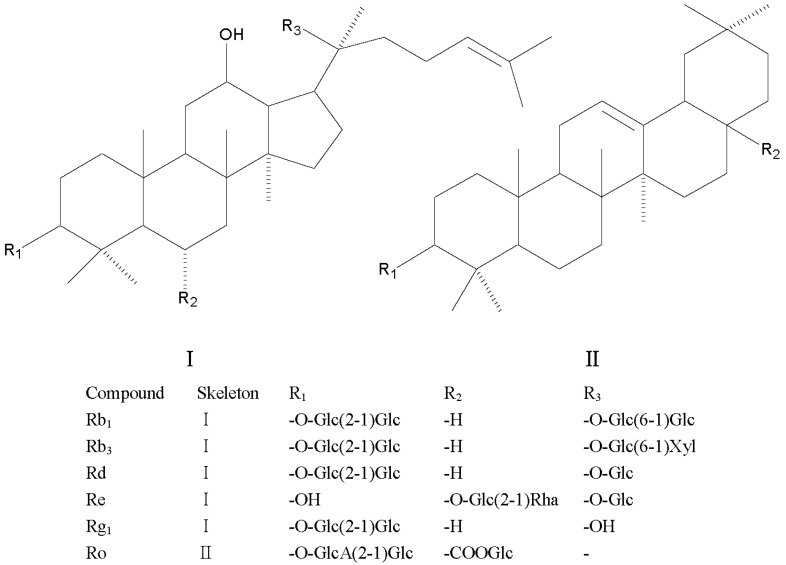
Chemical structures of ginsenosides.

The reference substances of seven aconitum alkaloids and six ginsenosides, namely BMA, BHA, BAC, MA, HA, AC, FZL, Rb_1_, Rb_3_, Rd, Re, Rg_1_, and Ro were purchased from Nanchang beta biotechnology Co., Ltd (Nanchang, China). Their chemical structures are shown in Figure 4 and Figure 5. respectively. The purities of these standard compounds were confirmed to be higher than 98% by HPLC analysis. Hongshen were purchased from Kangmei pharmaceutical Co.,Ltd (Guangzhou, China, 170904731) and Fuzi(Heishunpian) were purchased from Sichuan jiangyou zhongba Fuzi technology development Co., Ltd (Chengdu, China, 170502) and authenticated by professor Xiao-mei Fu (Jiangxi University of Traditional Chinese Medicine). Acetonitrile and methanol for analysis were MS grade and purchased from Merck (Darmstadt, Germany). Formic acid was HPLC grade and purchased from Dikma (Dikma, USA). Ultrapure water was obtained by a Milli-Q ultrapure water system (Millipore, Burlington, MA, USA).

### 3.2. Analytical System and Method for Qualitative Analysis

The qualitative analysis was performed on a Shimadzu UHPLC instrument coupled with a Triple-TOF 5600+ MS/MS system (AB SCIEX, Redwood, CA, USA) equipped with a DuoSpray™ Ion Source (shanghai, china). The separation was carried out on a Thermo Scientific (Waltham, MA, USA) Hypersil GOLD C18 column (100 mm × 2.1 mm, 1.9 μm) with 35 °C. Mobile phase was composed of 0.1% formic acid in water (A) and acetonitrile (B) with a gradient program as follows: 5% (B) in 0 to 2 min, 5% to 100% (B) in 2 to 30 min, 100% (B) in 30 to 32 min, 100% to 5% (B) in 32 to 35 min. The gradient elution was delivered at a flow rate of 0.2 mL/min. The injected volume was 2 µL. 

The mass spectra were acquired in positive and negative electron spray ionization (ESI) mode to provide comprehensive information for compound identification. Optimized parameters for positive and negative mode were as follows: The ion spray voltage, 5500 V (positive mode) and −4500 V (negative mode); declustering potential, 100 V (positive mode) and−100 V (negative mode); the turbo spray temperature, 600 °C (positive) and 500 °C (negative); the collision energy, 45 V (positive) and −45 V (negative). The collision energy spread was 15 V for both positive and negative mode. Nebulizer gas was N_2_ with Gas 1 (45 psi for positive and 40 psi for negative) and Gas 2 (heater gas, 45 psi for positive and 40 psi for negative). The curtain gas was kept at 30 psi. The mass range was scanned from 100 to 1500 *m*/*z* for parent ions and from 50 to 1500 *m*/*z* for daughter ions. 

Data acquisition and procession were carried out on Analyst 1.6 software and Peakview 2.2 software (AB SCIEX, Framingham, MA, USA).

### 3.3. Analytical System and Method for Quantitative Analysis

Quantitative analysis was performed on Shimadzu RRLC instrument coupled with a QTRAP 4500 system (AB SCIEX, Redwood, CA, USA); which was equipped with a binary high-pressure solvent delivery system (LC-30AD pump, Shimadzu Corporation, Kyoto, Japan). The separation was carried out on a Thermo Scientific Hypersil GOLD C18 column (100 mm × 2.1 mm, 1.9 μm) with 40 °C. Mobile phase was composed of 0.1% formic acid in water (A) and acetonitrile (B) with a fast gradient program as follows: 5% to 40% (B) in 0 to 3 min, 40% (B) in 3 to 5 min, 40% to 80% (B) in 5 to 5.5 min, 80% (B) in 5.5 to 7 min, 80% to 5% (B) in 7 to 7.1 min, 5% (B) in 7.1 to 9 min. The flow rate was set at 0.3 mL/min and the injection volume was 10 uL.

All analytes were confirmed and quantified by tandem mass spectrometry operating in electrospray positive ionization mode (ESI+) with MRM mode. The MS parameters were optimized and set as follows: Ion spray voltage at 5500 V, the turbo spray temperature at 500 °C, curtain gas (CUR) at 35 psi, nebulizer gas (GS1) at 50 psi, heater gas (GS2) at 50 psi, collision gas at 6 psi, and dwell time at 20 ms. The optimized declustering potential (DP) and proper collision energy (CE) are listed in Table 2.

Data acquisition and procession were performed on Analyst 1.6 software (AB SCIEX, Redwood, CA, USA). 

### 3.4. Preparation of Standard Solutions and Quality Control Solutions

The stock solutions of BMA, BHA, BAC, MA, HA, AC, FZL, Rb_1_, Rb_3_, Rd, Re, Rg_1_, and Ro were prepared in methanol at an accurate concentration of 1 mg/mL, respectively. A mixed stock solution was prepared by mixing appropriate aliquots of each stock solution together. Following this, a series of working solutions to the desired concentrations were achieved by doubling dilution with 50% methanol. Among of them, the high, medium, and low concentration solutions were selected as the quality control (QC) solutions for monitoring the status of system. All standard solutions were stored at 4 °C and were taken to room temperature before analysis. 

### 3.5. Preparation of Sample Solutions

Qualified Hongshen (S) and Fuzi (F) were mixed well for the preparation of five ShenFu prescriptions according to proper ratios. They were P1 (only Hongshen), P2 (S:F = 3:1, *w*/*w*), P3 (S:F = 1:1, *w*/*w*), P4 (S:F = 1:3, *w*/*w*), and P5 (only Fuzi), respectively. Decoctions were prepared by traditional decoction method. All the medicinal materials were soaked for 30 min beforehand. Fuzi were boiled for 1 hour firstly and then continually boiled or simmered together with Hongshen for three times [30]. The first time, 8 times the amount of water was added and decocted for 1 h. The second time, 6 times the amount of water was added and decocted for 45 min. The final time, water was added 2 to 3 cm above the residues and decocted for 30 min again. The decoctions were mixed together and concentrated to 1g/mL by rotary evaporation three times. All the samples were kept at 4 °C, diluted to a proper concentration and filtered through a 0.22 μm nylon membrane filter before analysis.

### 3.6. Validation of Method for Quantitative Analysis

The developed quantitative method was validated for linearity, LOD, LOQ, precision, repeatability, stability, and accuracy. A mixed working solution was diluted to seven appropriate concentrations and the linear curves for all analytes were constructed by plotting peak area (y) against concentration (x, ng/mL). The LODs and LOQs were obtained by diluting the mixed working solution to a very low concentration with signal-to-noise (S/N) ratios of 3 and 10, respectively.

Quality control (QC) (high, medium, and low concentrations) were analyzed six times in one day for intraday variations and examined in triplicate over three consecutive days for interday precision. To investigate the repeatability, six replicates of the same sample were prepared in parallel and analyzed. For stability testing, a sample solution was placed in an automatic sampler at 25 °C and analyzed at 0, 2, 4, 8, 12, and 24 h. All of the results were evaluated by relative standard deviations (RSDs) of the peak areas.

The recovery test for evaluating the accuracy of method was examined by adding three levels (80%, 100%, and 120% of the known amount) of the standard solutions to samples in triplicate. Recovery were calculated by the following formula: Recovery (%) = (detected amount − original amount)/spiked amount × 100. 

### 3.7. Sample Analysis

The chemical components of SFPD were investigated as a preliminary quality study by the qualitative analysis method. The contents of 13 analytes in five Shenfu prescriptions were determined as a further quality study by developed quantitative method. Moreover, the concept of solubilization ratio was employed to assess the compatibility effect of Hongshen and Fuzi. 

### 3.8. Establishment of an in-House Components Library of SFPD

Detailed and clear chemical constituents of SFPD are essential for holistic quality control. To ensure rapid and accurate identification of constituents in SFPD, an in-house constituent library that included the major known constituents of Hongshen and Fuzi was constructed by searching the databases of TCM Database @ Taiwan (http://tcm.cmu.edu.tw), TCMSP (Traditional Chinese Medicine Systems Pharmacology) Database (http://lsp.nwu.edu.cn/tcmsp.php), PubChem Database (http://www.ncbi.nlm.nih.gov/pccompound), MassBank (http://www.massbank.jp) Database, and so on.

## 4. Conclusions

In this study, taking SFPD as an example, a comprehensive and rapid strategy for quality evaluation and control of traditional Chinese medicine decoction was developed by integrating UPLC-QTOF-MS and UFLC-QQQ-MS technologies for qualitative and quantitative analysis, respectively. Consequently, a total of 39 compounds were tentatively identified, of which 21 compounds were unambiguously confirmed by comparing with reference substances. We determined 13 important constituents in SFPD within 7 min by MRM in positive ion mode. The developed quantitative method was employed for investigating the contents of different proportions of SFPD. The results indicated that the contents of 13 constituents did not increase or decrease proportionately in different ratio prescriptions, but generated solubilization effect or dissolution-inhibited effect, which was believed to be one of the main mechanisms of the synergistic effect of Hongshen and Fuzi. The mechanism of interaction effect of constituent dissolution is an important basis for the prescriptions of TCM. Nevertheless, more research should be designed to illustrate this further in the future. 

## Figures and Tables

**Table 1:** Identification of major compounds in SFPD by UPLC-QTOF-MS.

No	T_R_ (min)	Formula	Predicted (*m*/*z*)	Measured (*m*/*z*)	Mode	Error (ppm)	MS/MS(*m*/*z*)	Identification
1	1.32	C_10_H_13_NO_2_	180.1019	180.1016	[M + H]^+^	−1.6	180.1016, 115.0547, 145.0653	F/Salsolinol [23]
2	5.01	C_24_H_39_NO_9_	486.2698	486.2690	[M + H]^+^	−1.5	486.2690, 436.2320, 404.2064	F/Mesaconine [25]
3	5.38	C_22_H_35_NO_4_	378.2639	378.2639	[M + H]^+^	0.1	378.2639, 360.2524, 320.226	F/Aconosine [23]
4	5.41	C_22_H_35_NO_4_	378.2639	378.2639	[M + H]^+^	0.1	378.2639, 360.2524, 320.226	F/Karakoline [26]
5	5.46	C_23_H_37_NO_5_	408.2744	408.2744	[M + H]^+^	0	408.2744, 390.2635, 358.2383	F/Isotalatizidine [26]
6	5.72	C_22_H_31_NO_3_	358.2377	358.2379	[M + H]^+^	0.6	358.2380, 340.2271, 143.0866	F/Songorine [25]
7	5.81	C_25_H_41_NO_9_	500.2854	500.2852	[M + H]^+^	−0.4	500.2852, 450.2483, 468.2582	F/Aconine ^a^ [25]
8	6.48	C_24_H_39_NO_7_	454.2799	454.2796	[M + H]^+^	−0.8	454.2796, 436.2667, 404.2414	F/Fuziline ^a^ [25]
9	6.78	C_24_H_39_NO_6_	438.2850	438.285	[M + H]^+^	0	438.2850, 420.2737, 388.2480	F/Neoline [25]
10	7.43	C_24_H_39_NO_5_	422.2901	422.2902	[M + H]^+^	0.3	422.2902, 390.2633, 358.2365	F/Talatisamine [25]
11	8.11	C_25_H_41_NO_6_	452.3007	452.3007	[M + H]^+^	0	452.3007, 420.2743, 388.2477	F/Chasmanine [26]
12	9.44	C_31_H_41_NO_11_	604.2752	604.2746	[M + H]^+^	−1	604.2747, 605.2779, 554.2438	F/Flavaconitine [23]
13	9.95	C_31_H_43_NO_10_	590.2959	590.2954	[M + H]^+^	−0.9	590.2954, 540.2555, 558.2661	F/Benzoylmesaconine ^a^ [25]
14	10.08	C_48_H_82_O_18_	945.5428	945.5428	[M − H]^−^	0	945.5452, 637.4329, 475.3798, 161.0468	S/Ginsenoside Re ^a^
15	10.11	C_42_H_72_O_14_	799.4849	799.4813	[M − H]^−^	−4.5	799.4813, 637.4277, 475.3768	S/Ginsenoside Rg1 ^a^
16	10.18	C_36_H_60_O_8_	621.4361	621.4344	[M + H]^+^	−2.7	621.4344, 423.3623, 187.1478	S/Ginsenoside Rh4 [26]
17	10.18	C_15_H_24_O	221.1890	221.1899	[M + H]^+^	−0.5	221.1891	S/Spathulenol [23]
18	10.6	C_32_H_45_NO_10_	604.3116	604.3102	[M + H]^+^	−2.3	604.3100, 572.2832, 554.2722	F/Benzoylaconine ^a^ [25]
19	10.97	C_31_H_43_NO_9_	574.3011	574.3005	[M + H]^+^	−1	574.3005, 542.2727, 510.2492	F/Benzoylhypaconine ^a^ [26]
20	11.65	C_32_H_45_NO_9_	588.3167	588.3163	[M + H]^+^	−0.7	588.3163, 556.2896	F/Ludaconitine [23]
21	11.67	C_33_H_45_NO_12_	648.3014	648.3009	[M + H]^+^	−0.9	648.3007, 588.2791, 538.2441	F/Beiwutine [26]
22	12.41	C_42_H_72_O_14_	799.4849	799.4813	[M − H]^−^	−4.5	799.4815, 637.4277, 475.3768	S/Ginsenoside Rf ^a^
23	12.45	C_33_H_45_NO_11_	632.3065	632.3056	[M + H]^+^	−1.5	632.3056, 572.2839, 540.2585	F/Mesaconitine ^a^ [25]
24	12.69	C_54_H_92_O_23_	1107.5957	1107.5957	[M − H]^−^	0	1107.5980, 945.5447, 783.4916, 179.0565	S/Ginsenoside Rb1 ^a^
25	12.77	C_42_H_70_O_12_	767.4940	767.4929	[M + H]^+^	−1.5	767.4928, 605.4283, 163.0463	S/Ginsenoside Rg6 [26]
26	12.95	C_42_H_72_O_13_	783.4900	783.49	[M − H]^−^	0	783.4912, 637.4321, 475.3793, 161.0465	S/Ginsenoside Rg2^a^
27	13.02	C_36_H_62_O_9_	637.4321	637.4321	[M − H]^−^	0	637.4354, 475.3789, 391.2826, 101.0263, 71.0176	S/Ginsenoside Rh1 ^a^
28	13.09	C_42_H_72_O_13_	783.4900	783.4901	[M − H]^−^	0.1	783.4958, 637.4354, 475.3823, 161.0427	S/20(R)-Ginsenoside Rg2 ^a^
29	13.18	C_48_H_76_O_19_	955.4908	955.4908	[M − H]^−^	0	955.4846, 793.4326, 731.4332, 523.3753	S/Ginsenoside Ro ^a^
30	13.24	C_36_H_62_O_9_	637.4321	637.4321	[M − H]^−^	0	637.4397, 475.3806, 391.2703, 101.0252, 71.0195	S/20(R)-Ginsenoside Rh1 ^a^
31	13.3	C_33_H_45_NO_10_	616.3116	616.3110	[M + H]^+^	−1.1	616.3107, 556.2875, 524.2621, 338.1746	F/Hypaconitine ^a^ [25]
32	13.36	C_53_H_90_O_22_	1077.5851	1077.5852	[M − H]^-^	0	1077.5838, 945.5423, 621.4360, 149.0467	S/Ginsenoside Rb3 ^a^
33	13.37	C_34_H_47_NO_11_	646.3222	646.3214	[M + H]^+^	−1.3	646.3214, 586.3002, 526.2798	F/Aconitine ^a^ [25]
34	13.89	C_48_H_82_O_18_	945.5428	945.5428	[M − H]^−^	0	945.5498, 783.4960, 621.4398, 459.3865, 161.0473	S/Ginsenoside Rd ^a^
35	14.18	C_34_H_47_NO_10_	630.3273	630.3263	[M + H]^+^	−1.5	630.3259, 570.3069, 538.2808	F/Indaconitine [23]
36	14.27	C_34_H_47_NO_10_	630.3273	630.3263	[M + H]^+^	−1.5	630.3259, 570.3069, 538.2808	F/Deoxyaconitine [26]
37	16.94	C_42_H_72_O_13_	783.4900	783.4869	[M − H]^−^	−4	783.4865, 621.4342, 161.0478	S/20(R)-Ginsenoside Rg3 ^a^
38	17.03	C_42_H_72_O_13_	783.4900	783.4869	[M − H]^−^	−4	783.4865, 621.4342, 161.0478	S/Ginsenoside Rg3 ^a^
39	19.45	C_42_H_70_O_12_	765.4785	765.4795	[M − H]^−^	0.1	765.4769, 603.4240, 161.0462	S/Ginsenoside Rg5 [27]

^a^: The identity was confirmed by comparing the T_R_, MS/MS data with those of the reference substances. “F/” indicates that the components come from Fuzi, while “S/” indicates that the components come from Hongshen.

**Table 2:** Mass spectra properties of 13 analytes.

Analytes	Precursor Ion (*m*/*z*)	Product Ion (*m*/*z*)	DP. (V)	C.E. (V)
BMA	590.3	540.3	120	50
BHA	574.4	542.4	120	48
BAC	604.4	554.4	120	50
MA	632.2	572.4	100	47
HA	616.1	556.4	110	46
AC	646.2	586.2	120	47
FZL	454.3	436.5	130	43
Rb_1_	1131.7	365.1	135	44
Rb_3_	1101.5	789.4	250	70
Rd	969.6	789.6	209	66
Re	969.6	789.4	240	59
Rg_1_	823.5	643.5	162	54
Ro	979.6	845.6	263	70

**Table 3:** Regression equations, R^2^, linear ranges, (limit of detections) LODs and, limit of quantifications (LOQs) of 13 analytes.

Analytes	Regression Equation	R^2^	Linear Range (ng/mL)	LODs (ng/mL)	LOQs (ng/mL)
BMA	Y = 93571x + 51132	0.9985	0.05–50	0.003	0.01
BHA	Y = 147340x − 3680.9	0.9996	0.01–50	0.003	0.01
BAC	Y = 86804x + 45695	0.9941	0.01–50	0.003	0.01
MA	Y = 540315x + 116152	0.9969	0.01–25	0.003	0.01
HA	Y = 167089x + 75202	0.9962	0.01–50	0.003	0.01
AC	Y = 197019x + 70899	0.9988	0.01–50	0.003	0.01
FZL	Y = 165582x + 39050	0.9956	0.01–25	0.003	0.01
Rb_1_	Y = 17.256x + 143	0.9914	2.5–312.5	1.0	2.5
Rb_3_	Y = 537.803x − 1144	0.9954	2.5–312.5	1.0	2.5
Rd	Y = 9.8441x − 860.25	0.9951	25–6250	1.0	2.5
Re	Y = 945.03x + 4576.5	0.9927	2.5–156.2	1.0	2.5
Rg_1_	Y = 554.14x + 6673.9	0.9911	2.5–312.5	1.0	2.5
Ro	Y = 6.7504x + 5.3086	0.9956	5.0–312.5	1.0	2.5

**Table 4:** Precision, stability, and reproducibility of 13 analytes.

Analytes	Precision RSD%						Stability RSD% (*n* = 6)	Reproducibility RSD% (*n* = 6)
	Intra-Day (*n* = 6)			Inter-Day (*n* = 3)				
	Low	Medium	High	Low	Medium	High		
BMA	3.49	2.61	2.16	3.64	2.22	2.74	2.68	7.06
BHA	2.30	2.53	3.64	1.01	0.48	0.64	3.35	7.50
BAC	5.48	2.41	3.14	1.95	1.24	0.71	5.64	7.92
MA	3.39	3.16	2.49	2.11	2.97	3.65	2.43	8.59
HA	2.49	2.24	0.96	2.98	2.89	4.02	1.84	8.69
AC	2.15	2.00	1.23	3.86	1.44	2.95	4.33	9.14
FZL	6.45	5.91	3.82	3.22	1.36	1.72	2.12	9.18
Rb_1_	5.98	5.83	6.87	6.60	2.61	10.93	7.35	9.15
Rb_3_	3.89	1.32	2.99	9.90	7.53	5.09	5.28	9.75
Rd	7.55	2.59	1.66	8.20	4.06	7.24	2.54	7.88
Re	6.67	2.20	1.14	4.65	9.21	5.65	1.54	6.60
Rg_1_	6.47	5.47	3.56	7.40	6.45	5.60	3.89	7.55
Ro	6.41	6.25	6.49	6.67	9.33	8.58	6.75	10.13

**Table 5:** Recovery of 13 analytes.

Analytes	Initial Amount (ng)	Added Amount (ng)	Detected Amount (ng) (±SD, *n* = 3)	Recovery (%) (±SD, *n* = 9)	RSD (%) (*n* = 9)
BMA	2526.89	2000	4454.17 ± 21.99	98.19 ± 2.85	2.90
		2500	5003.63 ± 81.03		
		3000	5500.65 ± 109.08		
BHA	185.84	150	333.6 ± 11.34	99.82 ± 4.43	4.44
		185	373.27 ± 4.76		
		230	414.97 ± 6.76		
BAC	341.11	270	602.84 ± 5.73	98.95 ± 5.02	5.08
		340	679.52 ± 10.23		
		400	742.68 ± 35.26		
MA	27.88	20	46.81 ± 0.56	95.9 ± 3.62	3.77
		27	54.27 ± 1.48		
		35	61.26 ± 0.9		
HA	420.34	340	752.58 ± 19.9	99.2 ± 5.22	5.26
		420	831.73 ± 9.64		
		500	930.05 ± 36.2		
AC	22.76	18	40.75 ± 1.23	99.68 ± 6.06	6.08
		22	43.99 ± 1.46		
		28	51.47 ± 1.5		
FZL	686.25	550	1238.5 ± 39.48	99.57 ± 6.97	7.00
		680	1329.98 ± 37.27		
		820	1536.12 ± 58.53		
Rb_1_	3480.76	2700	6232.11 ± 238.45	103.89 ± 5.48	5.28
		3500	7097.51 ± 175.27		
		4200	7950.55 ± 42.83		
Rb_3_^a^	22.50	18	39.37 ± 0.25	97.37 ± 4.45	4.57
		22.5	44.52 ± 1.15		
		27	49.64 ± 1.07		
Rd ^a^	1283.56	1020	2231.22 ± 22.83	97.18 ± 6.53	6.72
		1280	2510.78 ± 62.47		
		1540	2866.19 ± 124.04		
Re	1084.36	850	1999.41 ± 6.77	105.73 ± 5.68	5.37
		1100	2189.04 ± 86.46		
		1300	2502.82 ± 17.04		
Rg_1_	4165.08	3300	7236.81 ± 73.35	95.14 ± 3.47	3.64
		4200	8314.75 ± 26.67		
		5000	8842.18 ± 176.42		
Ro	261.16	210	482.18 ± 7.72	106.43 ± 3.9	3.66
		260	532.11 ± 11.07		
		310	601.64 ± 4.77		

^a^: The unit of weight was µg.

**Table 6:** The contents and solubilization ratios of 13 analytes by UPLC-QQQ-MS (mg/L).

Analyte	P1 (S) Detected Amount	P2 (S:F = 3:1)			P3 (S:F = 1:1)			P4 (S:F = 1:3)			P5 (F) DetectedAmount
		DetectedAmount	Theoretical Amount	Solubilization Ratio (%)	DetectedAmount	Theoretical Amount	Solubilization Ratio (%)	DetectedAmount	Theoretical Amount	Solubilization Ratio (%)	
BMA	-	56.41	43.91	28.47	107.76	87.82	22.71	180.45	131.72	36.99	175.63
BHA	-	3.83	2.81	36.54	7.01	5.61	24.96	11.74	8.42	39.51	11.22
BAC	-	1.70	3.61	−52.84	9.35	7.21	29.68	16.35	10.82	51.18	14.42
MA	-	-	0.72	-	2.48	1.45	71.63	3.71	2.17	71.16	2.89
HA	-	3.49	4.56	−23.38	12.40	9.11	36.11	21.10	13.67	54.41	18.22
AC	-	-	-	-	0.82	-	-	0.75	-	-	-
FZL	-	9.24	8.03	15.07	20.48	16.06	27.52	35.42	24.09	47.03	32.12
Rb_1_^a^	4.14	3.65	3.11	17.38	1.50	1.82	−17.95	1.01	0.91	10.32	-
Rb_3_^a^	0.73	1.12	0.55	103.94	0.53	0.56	−5.14	0.36	0.28	29.57	-
Rd ^a^	71.40	70.71	53.55	32.05	27.30	35.36	−22.78	24.73	17.68	39.9	-
Re ^a^	2.71	2.48	2.03	22.16	0.88	1.24	−29.06	1.00	0.62	60.69	-
Rg_1_^a^	0.93	0.80	0.70	15.71	0.31	0.40	−21.76	0.36	0.20	76.94	-
Ro ^a^	0.42	0.25	0.32	−21.61	0.25	0.12	100.57	0.15	0.06	139.25	-

^a^: The unit of content is g/L.

## Supplementary Materials

The following are available online. Figure S1. Representative total ion chromatograms of the SFPD by UPLC-QTOF-MS. (A): TIC of P3 (S:F = 1:1) in positive ion mode; (B): TIC of P3 (S:F = 1:1) in negative ion mode; (C): TIC of P1 (S) in positive ion mode; (D): TIC of P1 (S) in negative ion mode; (E): TIC of P2 (S:F = 3:1) in positive ion mode; (F): TIC of P2 (S:F = 3:1) in negative ion mode; G: TIC of P4 (S:F = 1:3) in positive ion mode; H: TIC of P4 (S:F = 1:3) in negative ion mode; I: TIC of P5 (F) in positive ion mode; J: TIC of P5 (F) in negative ion mode.
