# Supplementary material for: A Comprehensive and Rapid Quality Evaluation Method of Traditional Chinese Medicine Decoction by Integrating UPLC-QTOF-MS and UFLC-QQQ-MS and Its Application
Source: Molecules. 2019 Jan 21;24(2):374. doi: 10.3390/molecules24020374 (PMC6359386; doi:10.3390/molecules24020374)
Supplement: Supplementary file 1 [file molecules-24-00374-s001.pdf]

Supplementary Materials:

A comprehensive and rapid quality evaluation method of traditional chinese medicine decoction by integrating UPLC-QTOF-MS and UFLC-QQQ-MS and its application.

Yinfang Chen, Riyue Yu, Li Jiang, Qiyun Zhang, Bingtao Li, Hongning Liu and Guoliang Xu

A (ESI+)_P3(S:F=1:1)

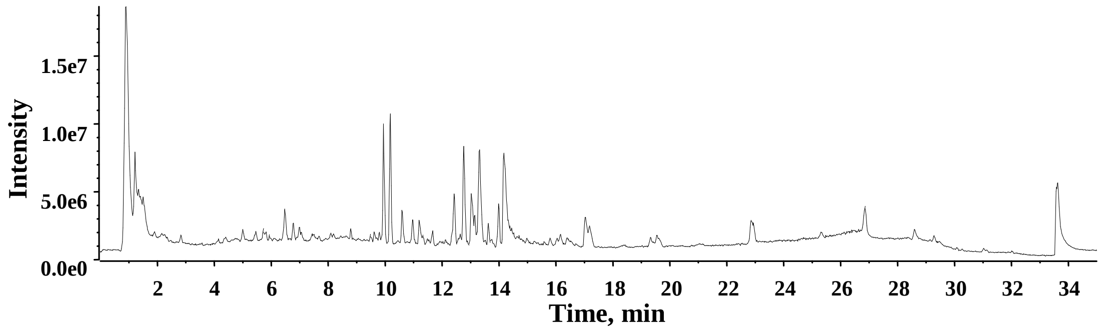

B (ESI-)_P3(S:F=1:1)

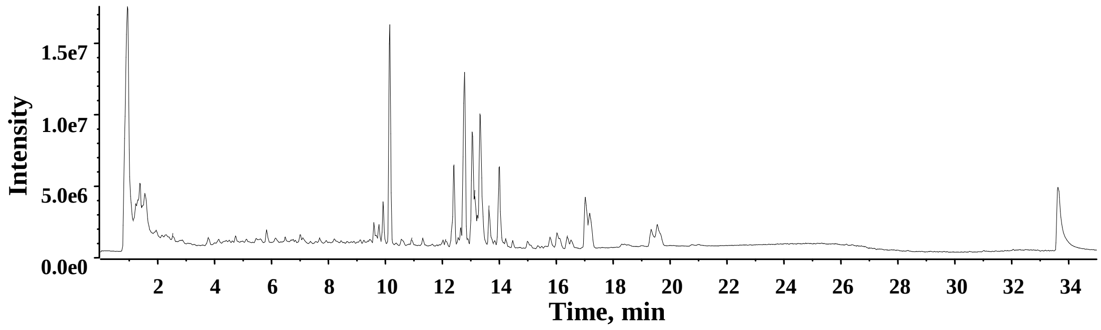

C (ESI+)_P1(S)

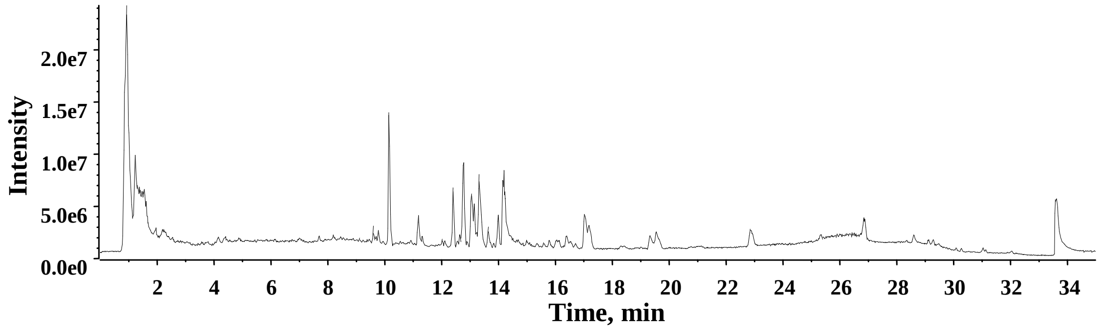

D (ESI-)_P1(S)

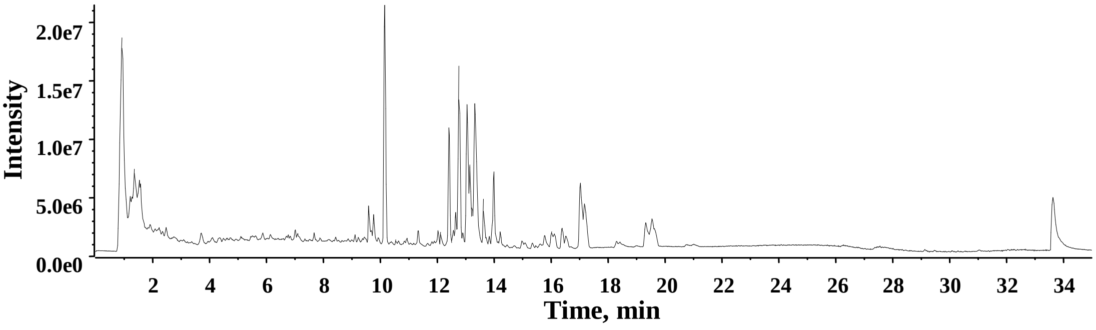

E (ESI+)_P2(S:F=3:1)

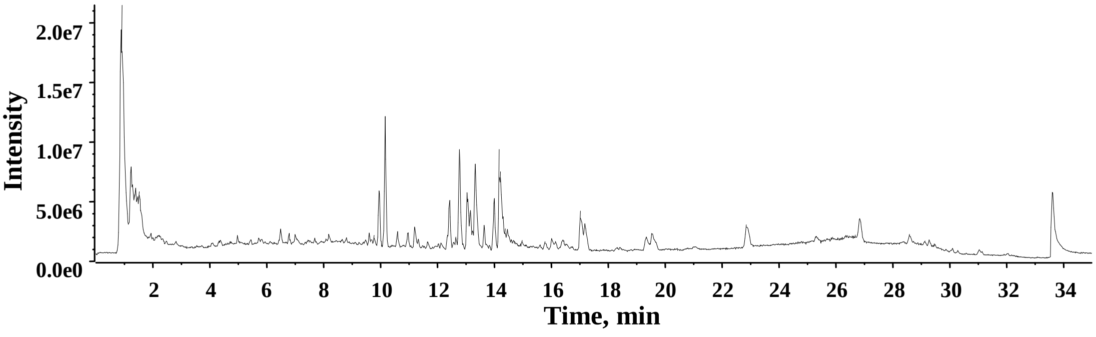

F (ESI-)_P2(S:F=3:1)

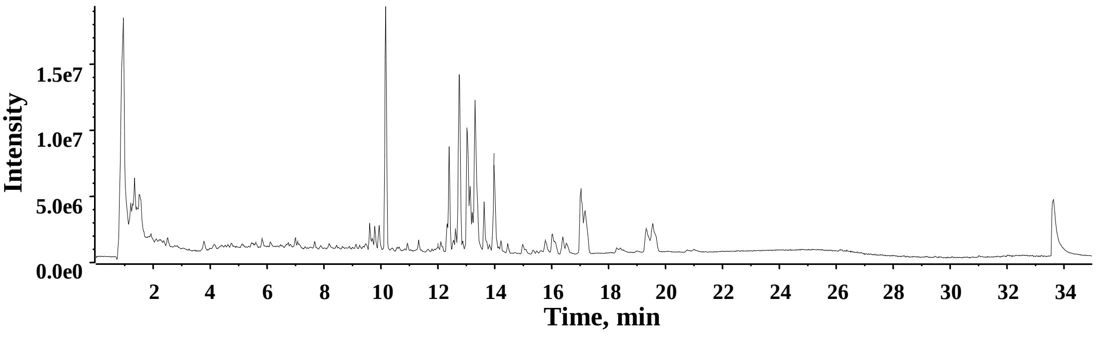

G (ESI+)_P4(S:F=1:3)

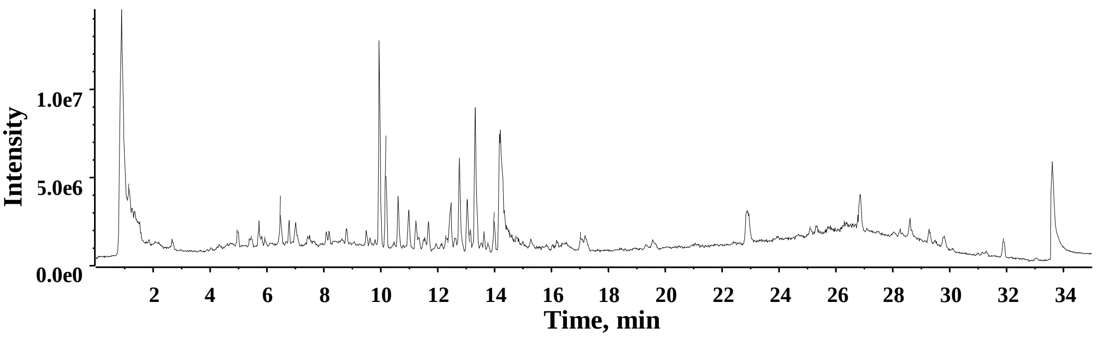

H (ESI-)_P4(S:F=1:3)

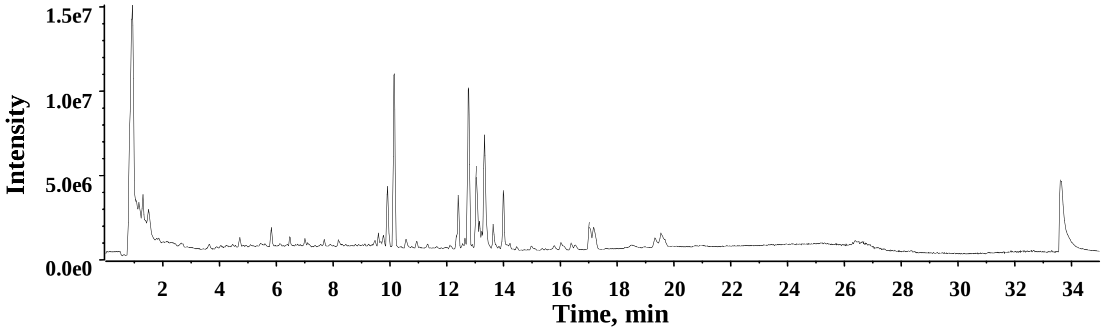

I (ESI+)_P5(F)

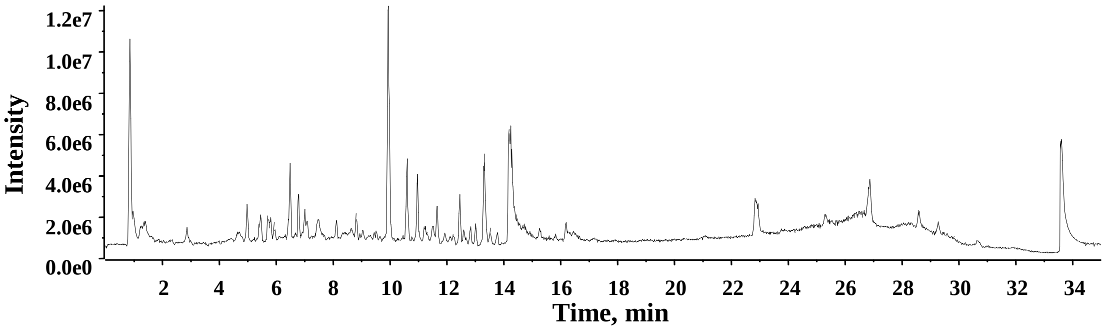

J (ESI-)_P5(F)

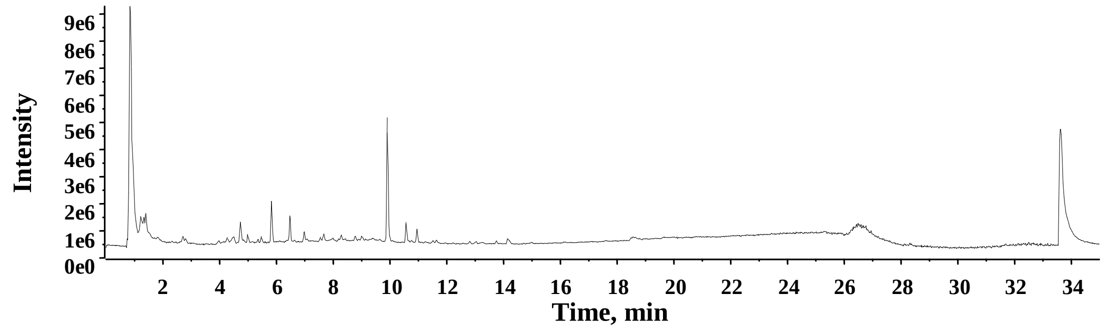

Figure S1. Representative total ion chromatograms of the SFPD by UPLC-QTOF-MS.
A: TIC of P3(S:F=1:1) in positive ion mode; B: TIC of P3(S:F=1:1) in negative ion mode; C: TIC of P1(S) in positive ion mode; D: TIC of P1(S) in negative ion mode; E: TIC of P2(S:F=3:1) in positive ion mode; F: TIC of P2(S:F=3:1) in negative ion mode; G: TIC of P4(S:F=1:3) in positive ion mode; H: TIC of P4(S:F=1:3) in negative ion mode; I: TIC of P5(F) in positive ion mode; J: TIC of P5(F) in negative ion mode.
